# Feasibility of fractionated gamma knife radiosurgery in the management of newly diagnosed Glioblastoma

**DOI:** 10.1186/s12885-022-10162-w

**Published:** 2022-10-26

**Authors:** Matthew Gallitto, Michelle Savacool, Albert Lee, Tony J. C. Wang, Michael B. Sisti

**Affiliations:** 1 Department of Radiation Oncology, Columbia University Irving Medical Center, 10032 New York, NY USA; 2 Department of Neurological Surgery, Columbia University Irving Medical Center, 10032 New York, NY USA

**Keywords:** Frameless stereotactic radiosurgery, Gamma Knife, Gamma Knife ICON, Glioblastoma, Hypofractionation, Radiosurgery

## Abstract

**Background:**

Glioblastoma (GBM) is the most common primary malignant brain tumor in adults, with overall survival remaining poor despite ongoing efforts to explore new treatment paradigms. Given these outcomes, efforts have been made to shorten treatment time. Recent data report on the safety of CyberKnife (CK) fractionated stereotactic radiosurgery (SRS) in the management of GBM using a five-fraction regimen. The latest Gamma Knife (GK) model also supports frameless SRS, and outcomes using GK SRS in the management of primary GBM have not yet been reported.

**Objective:**

To report on the feasibility of five-fraction SRS with the GammaKnife ICON in the management of newly diagnosed GBM.

**Methods:**

In this single institutional study, we retrospectively reviewed all patients from our medical center from January 2017 through December 2021 who received fractionated SRS with Gamma Knife ICON for newly diagnosed GBM. Patient demographics, upfront surgical margins, molecular subtyping, radiation treatment volumes, systemic therapies, and follow-up imaging findings were extracted to report on oncologic outcomes.

**Results:**

We identified six patients treated within the above time frame. Median age at diagnosis was 73.5 years, 66% were male, and had a median Karnofsky Performance Status (KPS) of 70. All tumors were IDH wild-type, and all but one were MGMT methylated and received concurrent temozolomide (TMZ). Within this group, progression free survival was comparable to that of historical data without significant radiation-induced toxicities.

**Conclusion:**

Gamma Knife ICON may be discussed as a potential treatment option for select GBM patients and warrants further investigation in the prospective setting.

## Introduction

Glioblastoma (GBM) is the most common primary malignant brain tumor in adults [1]. Despite ongoing efforts to explore more efficacious treatments, overall survival remains poor with a median survival time of 15 months and 5-year survival of ~ 5% after initial diagnosis [2]. Standard therapy is maximal safe surgical resection followed by radiation therapy to a dose of 60 Gy (Gy) in 30 fractions (fx) with concurrent and adjuvant temozolomide (TMZ) [3] with tumor treating fields [4]. Early trials establishing standard of care for GBM included only young patients with good performance status. With knowledge that the highest incidence of GBM is observed in those 75 to 84 years old [5], and elderly/frail individuals fare worse than younger and healthier patients with a median survival time of approximately 6 months [6–8], efforts have been made to shorten treatment time to increase treatment compliance and afford patients the opportunity to spend more time outside of treatment centers during the remainder of life. Several studies have demonstrated the efficacy of hypofractionated, shortened radiotherapy regimens for select older patients and/or those with poor performance status. In 2004, a prospective trial randomized 100 patients ages 60 and older after surgery to a radiation dose of 60 Gy in 30 fx versus 40 Gy in 15 fx without chemotherapy. Median survival was 5.1 months versus 5.6 months for standard and shorter course radiation, respectively [9]. The authors concluded that in patients older than 60 who are not receiving systemic therapy, there is no difference in overall survival when shortening adjuvant radiation therapy to a three-week course. *Perry et al.* later published results from a randomized trial concluding that in elderly patients with GBM, the addition of TMZ to short-course radiotherapy resulted in longer survival than short-course radiotherapy alone [10].

A more recent phase III non-inferiority trial randomized 98 GBM patients with poor performance status / advanced age to either an even shorter five-fraction treatment regimen (25 Gy in 5 fx) versus 40 Gy in 15 fx [11]. There were no differences in overall and progression-free survival. Furthermore, quality of life measures did not differ between patients receiving the two regimens. In this study, patients were treated on megavoltage equipment, cobalt-60 or linear accelerators. Single fraction stereotactic radiosurgery (SRS) was not permissible, MRI-based treatment planning was not required, and immobilization techniques / image-guidance parameters were not definitively outlined.

SRS utilizes highly precise radiation techniques to allow dose escalation and delivery of ablative doses to the tumor while minimizing dose to the adjacent normal tissue [12]. CyberKnife (CK) was developed as an image-guided frameless robotic SRS system. A lightweight linear accelerator is mounted on a robotic arm, making the CK capable of non-isocentric treatment. The latest version’s larger allowable field size conforms better to large irregularly shaped lesions, allowing for significant reductions in number of beams and treatment time. ICON, the latest version of Gamma Knife (GK), also supports frameless SRS with the use of cone-beam CT (CBCT), permitting stereotactic coordinate definition and daily positioning [13]. The ICON also incorporates an infrared camera-based intrafraction motion management (IFMM) system, allowing a thermoplastic mask to be used for immobilization without the need for an invasive metal frame. Planning can be done in advance and multiple fractions can be delivered quite efficiently [14].

In a recently published phase I/II trial of 5-fraction SRS with concurrent TMZ using CK, newly diagnosed GBM patients after surgery received 25–40 Gy in a dose-escalation fashion to determine the maximum tolerated dose (MTD). Authors found that the MTD of 5-fraction SRS with concurrent TMZ was 40 Gy in 5 fx, with limited grade 1–2 toxicities that did not statistically impact survival [15]. Several dosimetric analyses have shown GK is a superior modality for brain SRS owing to the ability to spare more normal brain tissue with better dose fall-off, and GK has a flexible workflow with physician-led forward treatment planning [13, 16]. In this study we report on our institutional experience of the feasibility of fractionated SRS with the Gamma Knife ICON in the management of newly diagnosed GBM.

## Methods

The protocol for this study was approved by our Institutional Review Board. We retrospectively reviewed all patients from our center from January 2017 through December 2021 who received fractionated SRS with Gamma Knife ICON for newly diagnosed primary GBM. ^14^After upfront biopsy and/or maximal surgical debulking, patients were selected for fractionated GK SRS after a multidisciplinary discussion with input from our institution’s neurosurgeons, radiation oncologists, and neuro-oncologists. All patients underwent magnetic resonance imaging (MRI) within 24 h after surgery/biopsy intended for frameless GKRS planning. 1 mm, thin-slice, volumetric, axial images were acquired down to C3 vertebral body, and T2 and T1 contrast-enhanced images were used for treatment planning. MRI images were imported into GammaPlan version 11.1.1, and the scalp border was defined. Both the radiation oncologist and the neurosurgeon participated in treatment planning. The post-operative resection bed and corresponding contrast enhancement in addition to residual gross disease was delineated as the gross tumor volume (GTV) on the MRI T1 contrast-enhanced image sequence. The clinical target volume (CTV) included a 1-1.5 cm margin respecting anatomical boundaries beyond the GTV to encompass microscopic disease, similar to other well-established hypofractionated radiation contouring guidelines [10]. The final planning target volume (PTV) included a 2 mm margin, with a prescription dose of 15–25 Gy to the 50-60% isodose lines in five daily fractions at the discretion of the treating radiation oncologist and neurosurgeon.

After treatment planning, patient setup and treatment delivery occurred in the ICON GK suite as previously described at our institution [14]. Briefly, while patient is in the supine position we molded a warmed thermoplastic mask over the patient’s face, folding back a rim of the mask around nasal aperture to prevent sharp edges. We then deployed the IFMM camera and placed the circular reflective marker on the patient’s nose for real-time intrafraction motion monitoring. CBCT was performed and registered with the planning MRI using Gamma Plan’s registration algorithm. Once the MRI-CBCT registration was complete, the dose distribution was recalculated to reflect the patient’s actual treatment position and geometry as defined by the reference CBCT. The updated dose distribution was reviewed, and modifications to the plan were made, if necessary, to ensure adequate dose to the target and acceptable dose sparing to the organs at risk. once the final distribution and treatment plans were approved, a second CBCT was performed for localization and registered to the reference CBCT. An updated dose distribution and dose-volume histogram (DVH) reflecting the patient geometry in the pretreatment CBCT was reviewed and if satisfactory, the treatment was delivered. Subsequent fractions required only one pretreatment CBCT for localization. Intrafraction monitoring with the IFMM is set to allow nasal tip motion of up to 3 mm during treatment, however this tolerance may be reduced at the discretion of the treating physician. Deviation beyond the threshold for > 30 s automatically aborts radiation delivery, removes patient from the GK bore, and a repeat localization CBCT is required before treatment can resume. After completion of radiation therapy, patients followed closely with their primary neuro-oncologist, radiation oncologist, and neurosurgeon.

For this study, datapoints including patient demographics, surgical margins, molecular subtyping, radiation treatment volumes, systemic therapies, and follow-up imaging findings were extracted from the electronic medical record. Median follow-up time, median PFS, median OS, and other descriptive statistics for this patient cohort were calculated using GraphPad Prism 7.0.

## Results

Between January 2017 and December 2021, we identified six patients with newly diagnosed GBM treated with fractionated SRS using the Gamma Knife ICON. Median age at diagnosis was 73.5 years (range 47–82), 66% were male, and had a median Karnofsky Performance Status (KPS) of 70 (range 60–80). All patients were IDH wild-type on immunohistochemical staining. Using primary surgical specimen, DNA was bisulfite treated and amplified with PCR primers flanking the first 21 CpGs of the MGMT promoter CpG island, followed by melting curve analysis to distinguish the methylated from unmethylated allele. All patients except one were MGMT methylated. Median time from surgery/biopsy to initiation and completion of radiosurgery was 17 days (range 7–27) and 22 days (range 13–33), respectively. Of note, two patients had gross total resections based on post-operative MRI, and three patients had subtotal resections. All but one patient received concurrent TMZ with radiation therapy, and all patients received at least 1 cycle of adjuvant TMZ. Dose was prescribed as 25 Gy to the 60% isodose line in five fractions to all patients, except one patient received 15 Gy to the 50% isodose line in 5 fractions. Median planning treatment volume (PTV) was 87 cc (range 20–147), which included the 2 mm PTV margin. No acute radiation-related toxicities were noted in the treated patients.

Post-radiation treatment brain MRIs were conducted a median of 42 days (range 24–61) after completion of radiosurgery. Using the Response Evaluation Criteria in Solid Tumors (RECIST) criteria, two patients had a complete response, two patients had stable disease, and two patients had partial response. No patients had disease progression at the time of first post-treatment imaging. Median follow-up time was 8.0 months (range 6.5–55), and median PFS after upfront fractionated SRS was 5.6 months (range 3.1–36). Median OS was not reached as all except one patient remained alive at the time of chart review.

**Fig. 1:**
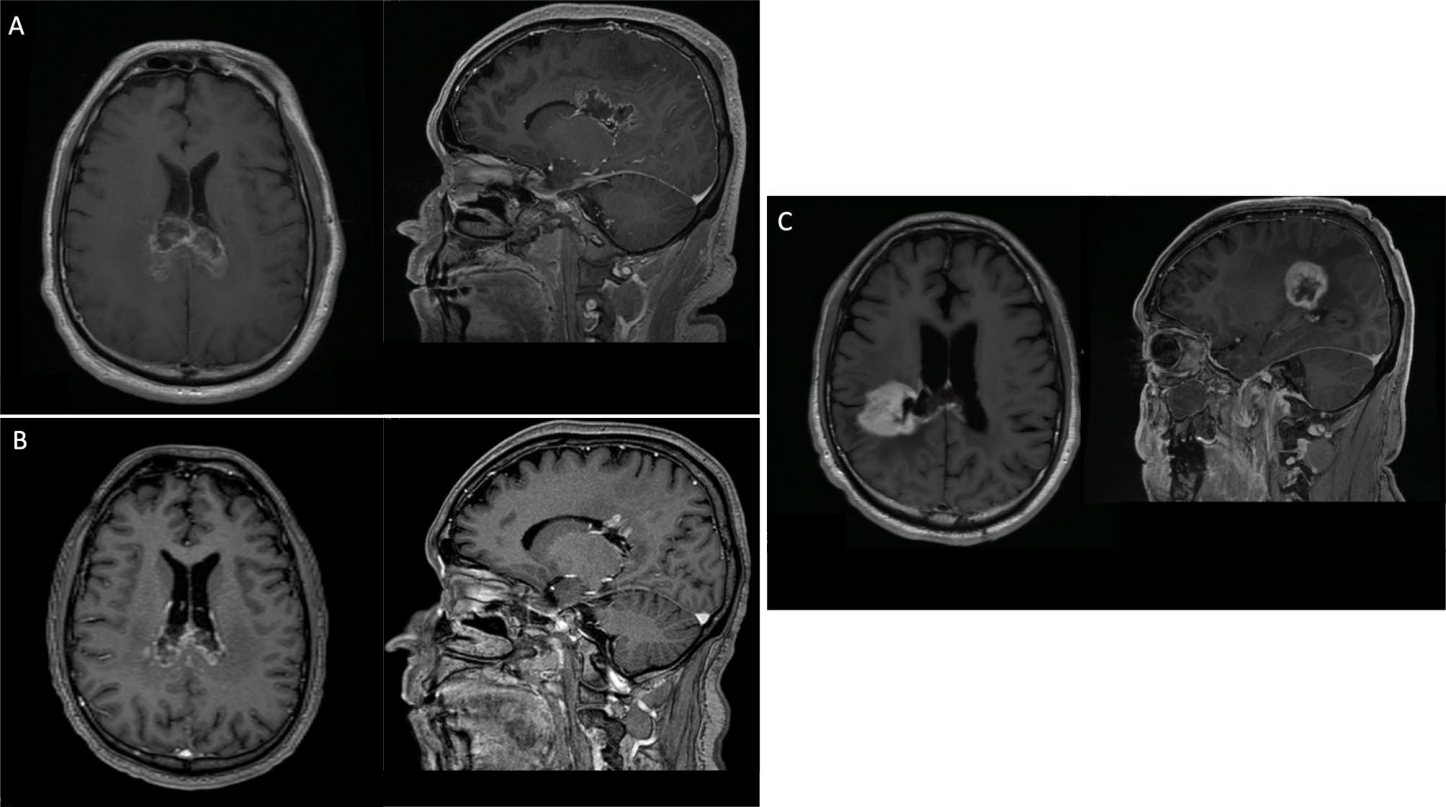
MRI brain T1-post contrast axial and sagittal views for a selected patient (A) 24-hours post-op prior to receiving radiosurgery with GammaKnife ICON, (B) one month after completion of radiation treatment showing a partial response per RECIST criteria, and (C) 3 years after completion of radiation treatment at time of radiographic evidence of disease progression

**Fig. 2:**
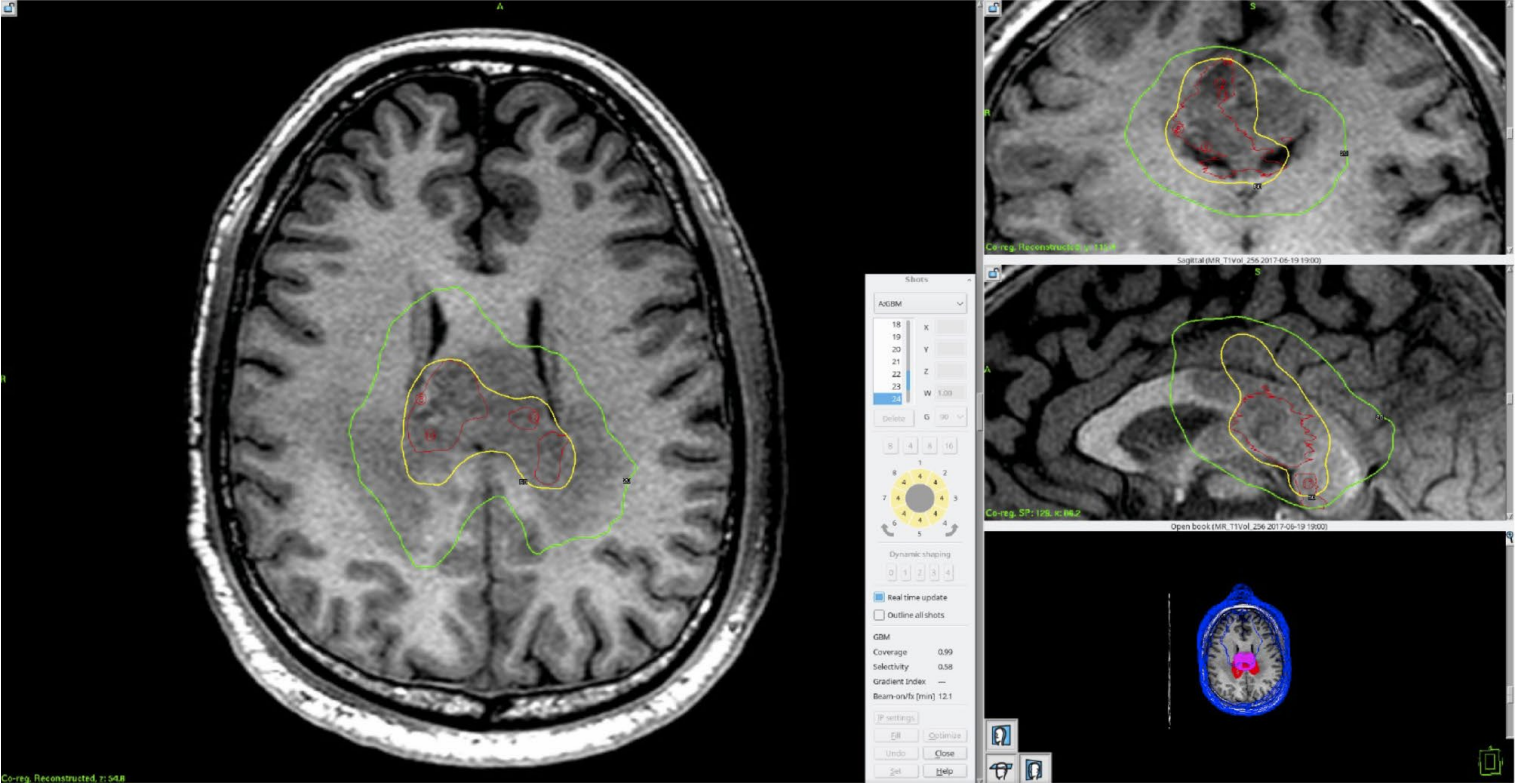
Screenshot obtained from GammaPlan version 11.1.1 treatment planning software containing volumetric T1-post contrast MRI brain images after upfront surgical debulking for a selected patient receiving fractionated radiosurgery with GammaKnife ICON. Outlined in red is the post-operative intended treatment volume as determined by fusion with pre-operative imaging as well as close collaboration with the neurosurgical team to determine residual disease versus post-operative changes (15.5 cc volume). Yellow and green represent the 50% and 20% isodose lines, respectively. Dose was prescribed to the 50% isodose line (yellow). As outlined, coverage of the treatment volume was 99% with a 58% selectivity and beam-on time of 12.1 min per fraction

Three patients were still without clinical or radiographic evidence of disease at 6.0 months, 7.6 months, and 7.0 months from the date of diagnosis. One patient showed radiographic evidence of recurrence at 3.1 months, with an overall survival of 10.0 months. Death was due to neurological disease progression. One patient showed radiographic evidence of disease recurrence at 5.6 months and is currently with stable disease at 10 months. Excitingly, one patient had a PFS of 3.0 years after completing concurrent and adjuvant TMZ for 12 cycles. At the time of recurrence, he underwent surgical debulking followed by bevacizumab and pembrolizumab. Carboplatin has been added to his systemic therapy regimen given slow interval progression of disease, but continues to be alive at 4.5 years post-diagnosis. For educational purposes, we provide the imaging characteristics and treatment planning for this patient in Figs. 1 and 2, respectively.

## Discussion

To our knowledge, this is the first study to report on the feasibility of fractionated SRS using the GammaKnife ICON in the management of newly diagnosed GBM. With a continued movement towards hypofractionated regimens in order to afford patients the opportunity to spend more of their remaining time outside treatment facilities, GammaKnife ICON appears to be a reasonable approach at our institution for select GBM patients. Traditionally, time from surgery to completion of adjuvant conventionally fractionated chemoradiation can routinely extend for greater than two months. This poses a significant burden to patients and their families. Based on this small retrospective analysis, using GammaKnife ICON can shorten this duration to significantly less than one month. This regimen may be of particular interest in those where outpatient daily transportation would be difficult to coordinate perhaps due to patient condition, or socioeconomic barriers including transportation and financial constraints.

It is important to highlight that treatment volumes are important to consider when deciding on the use of GammaKnife ICON. In patients with significantly large tumor volume or resection cavities, treatment times using this modality may be too long and therefore not feasible. Thus, discussion is warranted with the treating physician as well as physicist to ensure feasibility of this approach for each individual patient.

All patients in this study had IDH-wildtype tumors, and all except one exhibited MGMT promoter methylation. It is well known that MGMT promoter methylation is associated with improved outcome in GBM and is likely a predictive marker of sensitivity to alkylating agents. It is important to note that the one patient who had an unmethylated tumor was also the patient with the longest PFS of 3.0 years. This patient also did not receive concurrent TMZ with radiosurgery. In future studies, it will be important to understand the impact of MGMT methylation and the use of concurrent TMZ on outcomes in patients receiving this hypofractionated regimen using GammaKnife ICON.

Although our sample size is small, progression free survival appears comparable to historical data. Roa et al. reported a median PFS of 4.2 months using a similar hypofractionated dose regimen of 25 Gy in 5 fractions. In this trial, MRI-based treatment planning and image-guidance were not required. Furthermore, the tighter quality-assurance tolerances of SRS-based treatments were not required on this trial [11]. In our study, many patients have not yet had disease recurrence which limits our ability to provide more concrete times to progression and overall survival times.

## Limitations

In addition to short interval follow-up time, we recognize the other limitations of this small, retrospective single institutional analysis including selection bias. Further prospective randomized trials are necessary to provide more robust data. The purpose of this report is not to claim non-inferiority or equivalence of the GammaKnife ICON for primary GBM.

## Conclusion

As the first study to report on the experience with fractionated SRS using the GammaKnife ICON in the management of newly diagnosed GBM, it appears this treatment paradigm is safe and feasible with similar oncologic outcomes to historical controls without the burden of significant radiation-induced toxicities. Thus, in select patients where shortened treatment time are necessary, GammaKnife ICON may be discussed as an option with patients and families in a multidisciplinary setting.

## Data Availability

The datasets analyzed during the study are not publicly available to protect patient privacy. However, minimal dataset that would be necessary to interpret, replicate and build upon the findings reported are available from the corresponding author on reasonable request.

## References

[1] Ostrom QT (2015). CBTRUS Statistical Report: Primary Brain and Central Nervous System Tumors Diagnosed in the United States in 2008–2012. Neuro Oncol.

[2] Chang K (2016). Multimodal imaging patterns predict survival in recurrent glioblastoma patients treated with bevacizumab. Neuro Oncol.

[3] Stupp R (2005). Radiotherapy plus concomitant and adjuvant temozolomide for glioblastoma. N Engl J Med.

[4] Stupp R (2017). Effect of Tumor-Treating Fields Plus Maintenance Temozolomide vs Maintenance Temozolomide Alone on Survival in Patients With Glioblastoma: A Randomized Clinical Trial. JAMA.

[5] Dolecek TA, Propp JM, Stroup NE, Kruchko C (2012). CBTRUS statistical report: primary brain and central nervous system tumors diagnosed in the United States in 2005–2009. Neuro Oncol.

[6] Iwamoto FM, Reiner AS, Panageas KS, Elkin EB, Abrey L (2008). E. Patterns of care in elderly glioblastoma patients. Ann Neurol.

[7] Kita D (2009). Age as a predictive factor in glioblastomas: population-based study. Neuroepidemiology.

[8] Paszat L (2001). A population-based study of glioblastoma multiforme. Int J Radiat Oncol Biol Phys.

[9] Roa W (2004). Abbreviated course of radiation therapy in older patients with glioblastoma multiforme: a prospective randomized clinical trial. J Clin Oncol.

[10] Perry JR (2017). Short-Course Radiation plus Temozolomide in Elderly Patients with Glioblastoma. N Engl J Med.

[11] Roa W (2015). International Atomic Energy Agency Randomized Phase III Study of Radiation Therapy in Elderly and/or Frail Patients With Newly Diagnosed Glioblastoma Multiforme. J Clin Oncol.

[12] Redmond KJ, Mehta M (2015). Stereotactic Radiosurgery for Glioblastoma. Cureus.

[13] Han EY, Wang H, Luo D, Li J, Wang X (2019). Dosimetric comparison of fractionated radiosurgery plans using frameless Gamma Knife ICON and CyberKnife systems with linear accelerator-based radiosurgery plans for multiple large brain metastases. J Neurosurg.

[14] Vulpe H (2020). Frameless Stereotactic Radiosurgery on the Gamma Knife Icon: Early Experience From 100 Patients. Neurosurgery.

[15] Azoulay M (2020). A phase I/II trial of 5-fraction stereotactic radiosurgery with 5-mm margins with concurrent temozolomide in newly diagnosed glioblastoma: primary outcomes. Neuro Oncol.

[16] Dong P (2016). Dosimetric characterization of hypofractionated Gamma Knife radiosurgery of large or complex brain tumors versus linear accelerator-based treatments. J Neurosurg.

